# 3D-CT Evaluation of Swallowing: Metrics of the Swallowing Response Using Swallowing CT

**DOI:** 10.1007/s00455-021-10288-2

**Published:** 2021-04-05

**Authors:** Yoko Inamoto, Marlís González-Fernández, Eiichi Saitoh

**Affiliations:** 1 Faculty of Rehabilitation, School of Health Sciences, Fujita Health University, 1-98 Dengakugakubo, Kutsukake, Toyoake, Aichi 470-1192 Japan; 2 Department of Rehabilitation Medicine I, School of Medicine, Fujita Health University, Toyoake, Aichi Japan; 3 Department of Physical Medicine and Rehabilitation and Orthopaedic Surgery, Johns Hopkins University School of Medicine, Baltimore, MD USA

**Keywords:** Swallowing, Computed tomography, Evaluation, Larynx, Pharynx, Upper esophageal sphincter, Rehabilitation, Deglutition, Deglutition disorders, Physiology, Pathophysiology

## Abstract

Videofluoroscopy and videoendoscopy dramatically changed the evaluation and management of swallowing disorders. Later advancements in techniques for the instrumental evaluation of swallowing were limited by technique and positioning. The advent of 320-row area detector CT solved previous challenges and allowed for the study of swallowing physiology and dysphagia in greater detail. In this summary, we describe the history and evolution of CT technology and describe research and clinical applications for the evaluation of swallowing physiology and pathophysiology.

## Introduction

Videofluoroscopy (VF) and videoendoscopy (VE) allow for real-time dynamic evaluation of swallowing. They are the main tools used for standard swallowing evaluation. VF allows the kinematic analyses essential for understanding swallowing physiology and the mechanisms resulting in dysphagia [1]. VE provides clinicians with a convenient bedside assessment of anatomical structures and pharyngeal physiology [2]. The ability to visualize abnormal findings such as aspiration and pharyngeal residue and characterize impairments during VF or VE informs optimal treatment strategies. For decades, they have been the primary tools for the clinical evaluation and management of dysphagia.

Computed tomography (CT), developed in 1970s, is an imaging modality that produces the cross-sectional images of the scanned structures. Large clinical demand and rapid increase in use have contributed to remarkable technological developments including rapid and wider scanning, (i.e., helical CT and cone-beam CT) and the development of three-dimensional visualization (multidetector CT). The application of CT for dysphagia assessment and rehabilitation started in the 1980s. However, its practical use was not possible until the development of a multidetector CT, such as 320-row multidetector CT (Aquilion ONE, Canon Medical, Japan). The 320-row multidetector CT is equipped with 320-rows of 0.5 mm detectors along the body axis and can scan a range of 16 cm in a single tube rotation with a shorter acquisition time [3]. Due to the wide range of scanning ability, 320-row multidetector CT scanners are also called 320-row area detector CT (320-ADCT). Dynamic swallowing evaluation became possible using this technology.

Another imaging modality for swallowing studies, magnetic resonance imaging (MRI), was used starting in the 1990s using echo-planar imaging, turbo-FLASH techniques, and single-shot fast spin echo techniques [4–7]. Three-Tesla MRI enabled high-speed imaging with temporal resolution of 24.3 frames per second and enabled the dynamic imaging of swallowing [8–11]. Despite the temporal resolution, restrictions to image acquisition (single plane acquisition) limit clinical application.

Since the first swallowing CT study using 320-ADCT published in 2011, dynamic 3D images and quantitative kinematic analysis have significantly expanded the understanding of oropharyngeal morphology, swallowing physiology, and kinematics. It has also been used in dysphagia rehabilitation for quantitative understanding of pathophysiology. In this paper, we review the utilization of CT technology for swallowing research. After a brief historical perspective, two early swallowing studies using the Electron Beam Computed Tomography and helical CT are reviewed. Then, studies of swallowing CT using 320-ADCT are reviewed including CT characteristics, research metrics, and morphological and kinematic findings. The majority of the CT studies reviewed are our team’s work thus this is not a systematic review.

## CT of Swallowing: A Historical Perspective

### History of CT (Fig. 1)

**Fig. 1:**
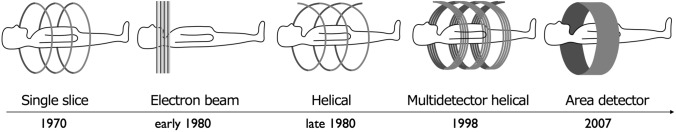
Development of CT

The first invented CT scanner was a single slice CT allowing for one slice data by a single detector to be acquired for each tube rotation [12]. To scan the target organ, a number of single scanning revolutions needs to be performed to obtain each slice, resulting in long scanning time and artifact. In the early 1980, Electron Beam Computed Tomography (EBCT) was developed [13]. Using a large stationary x-ray tube that partially surrounds the imaging field, tube rotation assembly was not necessary, resulting a reduced scan times to 50–100 ms (depending on the imaging mode) for each slice acquisition [13]. However, technical challenges such as poor quality images due to the limited X-ray power, limited the achievement of its widespread adoption in clinic. In the late 1980s, helical CT, or spiral CT scanners were developed and were rapidly adopted clinically. By scanning the body spirally, the target area can be evaluated in a shorter period allowing scanning time to be remarkably reduced [14]. Furthermore, to meet the clinical expectations of wider range scanning and three-dimensional image acquisition, in 1998, multi-slice CT, or multi-detector CT, was invented [15, 16]. Multi-slice CT has multiple rows of detectors, allowing data for multiple slices to be acquired simultaneously in each tube rotation [15]. It enables the acquisition of three-dimensional images by layering multiple slices using various reconstruction methods, such as multi-planar reconstruction (MPR) and volume rendering. Multi-slice CT allows the acquisition of three-dimensional data of the target body part quickly [15]. The number of the detector rows increased gradually from 2, 4, 8, 16, 64 to 256. In 2007, the number of detectors reached 320, corresponding to a total detector width of 16 cm [17]. Because a 16 cm range can cover the majority of human organs, scanning can be performed without helical rotation in the area of interest [17]. This repeated scanning allows for the acquisition of images over time or dynamic 3D-CT images (4D images; 3D × time).

### Dynamic Evaluation of Swallowing

**Fig. 2:**
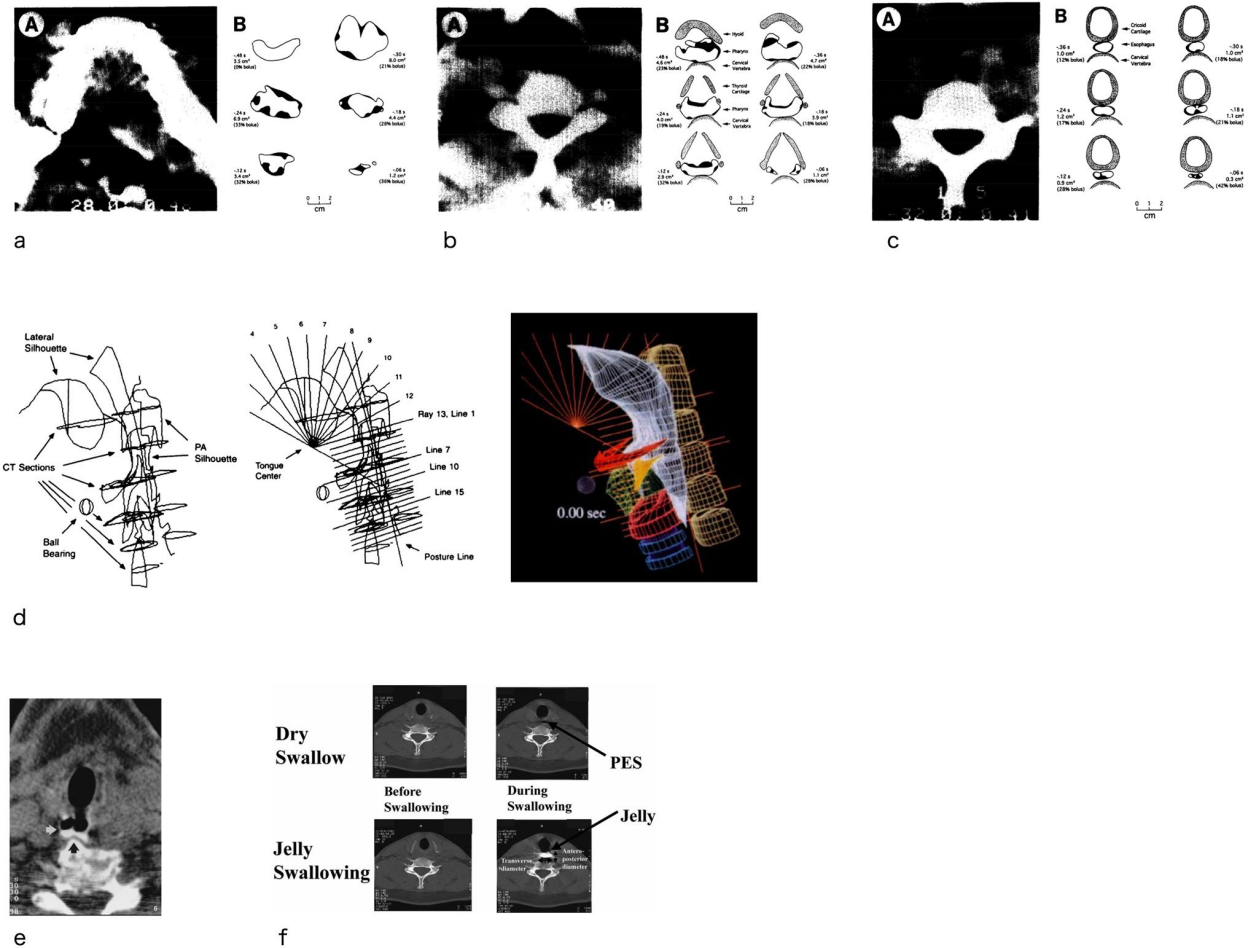
CT images from previous studies. Axial images acquired from Electron Beam Computed Tomography at the level of glossopalatal junction (**a**), at the level of valleclae (**b**), and at the level of UES (**c**), cited Figs. 2–4 of Ergun’s study [18]. **d** Three-dimensional modeling of oropharyngeal swallow using the images of synchronized EBCT and VF. Cited Figs. 2 and 3 of Kahrilas’s study [19]. **e** Axial images at the level of UES (white arrows), cited Fig. 6 of Lindbichler’s study [20]. **f** Axial images of PES before swallowing and during swallowing of dry swallow (upper) and jelly swallowing (lower), cited Fig. 2 of Takehara and Chu’s study [22]. Reproduced with permission from [18, 19, 20, 22]

A few studies were performed to study the dynamic movements of swallowing using Electron Beam computed tomography (EBCT, Imatron C-100 Ultrafast CT scan) [18–20]. In the early 1990s Ergun and colleagues utilized EBCT to describe the shape, volume, and content of the pharynx on axial images during swallowing [18]. Eight axial images with a slice thickness of 8 mm, generated with four-row X-ray target rings and adjacent two detectors, were obtained from four levels of the pharynx, starting at the glossopalatal region and proceeding caudally, encompassing a range of 8 cm of pharynx at a rate of 17.24 frames per second (1.5 s scanning time). Axial images at specific levels (glosspalatal region, tongue base, valleculae, hypopharynx, UES, proximal esophagus) were obtained (Fig. 2a–c) [18]. They described that the pharyngeal chamber was largest at the level of the vallecula, became smaller caudally, and was smallest at the UES, which was ovoid shaped [18]. Measurements of air content and bolus in each plane allowed the investigators to describe that, typically, less than 30% of the lumen was occupied by the bolus and to estimate that 15 ml of air are present in each area in addition to bolus volume [18]. The same group performed synchronized EBCT and VF scans of one healthy subject [19]. They aligned the images and constructed a dynamic 3D model of the oropharyngeal swallow using 3D graphics-animation software (Fig. 2d). Towards the end of the decade, Lindbicher et al. used EBCT to examine swallowing in three axial planes of the pharynx [20] (Fig. 2e). Each axial plane was scanned using a single-slice cine mode with scan times of 100 ms, interscan delay of 16 ms, in 2.3 s and 20 slices with a thickness of 3-mm or 6-mm. Due to the technical limitation of acquisition of a maximum 8 slices in the range of 8 cm, EBCT could not provide dynamic images of the whole pharynx during swallowing, but was limited to imaging in the transverse plane.

Helical CT, or spiral CT followed EBCT during the 1990s. Helical CT obtains images during a single breath-hold, reducing movement artifact and providing continuous volumetric acquisition as the patient moves through the gantry [21]. The applicability of helical CT to the study of swallowing was described by Takehara and Chu in 2004. They obtained sequential axial images of the pharynx and UES during bolus transport. Measurements of UES opening were possible (Fig. 2f). They reported larger anteroposterior and transverse diameter and area of the UES with larger bolus volume [22].

Both EBCT and helical CT provided some insights into swallowing physiology by providing the first visualization of the pharynx and UES in the axial plane. However, single plane images, image processing, supine position, and high radiation dose limited the clinical application of EBCT and helical CT in the evaluation of swallowing.

Several anatomical studies reported the length and volume of the pharyngeal airway by assessing shape with static imaged acquired by helical CT or Cone-Beam CT [23–25]. For example, the axial images obtained using serial CT in a case reported by Tsukamoto [25] allowed for the identification of the area of the pharynx that was closed during head rotation to the paralyzed side in dysphagia due to lateral medullary syndrome.

## Using 320-Row Area Detector CT

### Scanner Characteristics

Multidetector CT became available in the 1990s enabling the acquisition of 3D images. The utilization of multidetector CT was again considered in the study of swallowing: however, helical scanning does not allow for the acquisition of dynamic 3D-CT images. The study of swallowing became possible with 320-row area detector CT (Aquilion ONE, Toshiba medical—currently Canon Medical, Japan). This technology continues to be used in research and clinical applications to evaluate swallowing today, known typically as ‘Swallowing CT’.

#### Data Acquisition [3, 26]

A 16 cm range can cover the organs from the skull base to the upper esophagus which is necessary for swallowing assessment. By single-phase volume scanning (single volume scan) during which the tube is rotated once every 0.35 s, static 3D images of target structures within this 16 cm range are acquired. By multi-phase scanning (dynamic volume scan), the tube is rotated repeatedly (0.35 s × *X* rotations; 0.35 s times the selected number of rotations) thus dynamic 3D images and motion of target structures within this range are acquired.

Over the years, 320-ADCT technology has improved. The second generation of 320-ADCT (2011-Aquilion ONE Vision) allowed the tube rotation speed to increase from 0.35 to 0.275 s and decrease the radiation dose with the addition of adaptative iterative dose reduction 3D (AIDR 3D). The third generation (2015—Aquilion ONE Genesis), further decreased the radiation dose, and allowed for tube tilt to be increased from 22° to 30°.

**Fig. 3:**
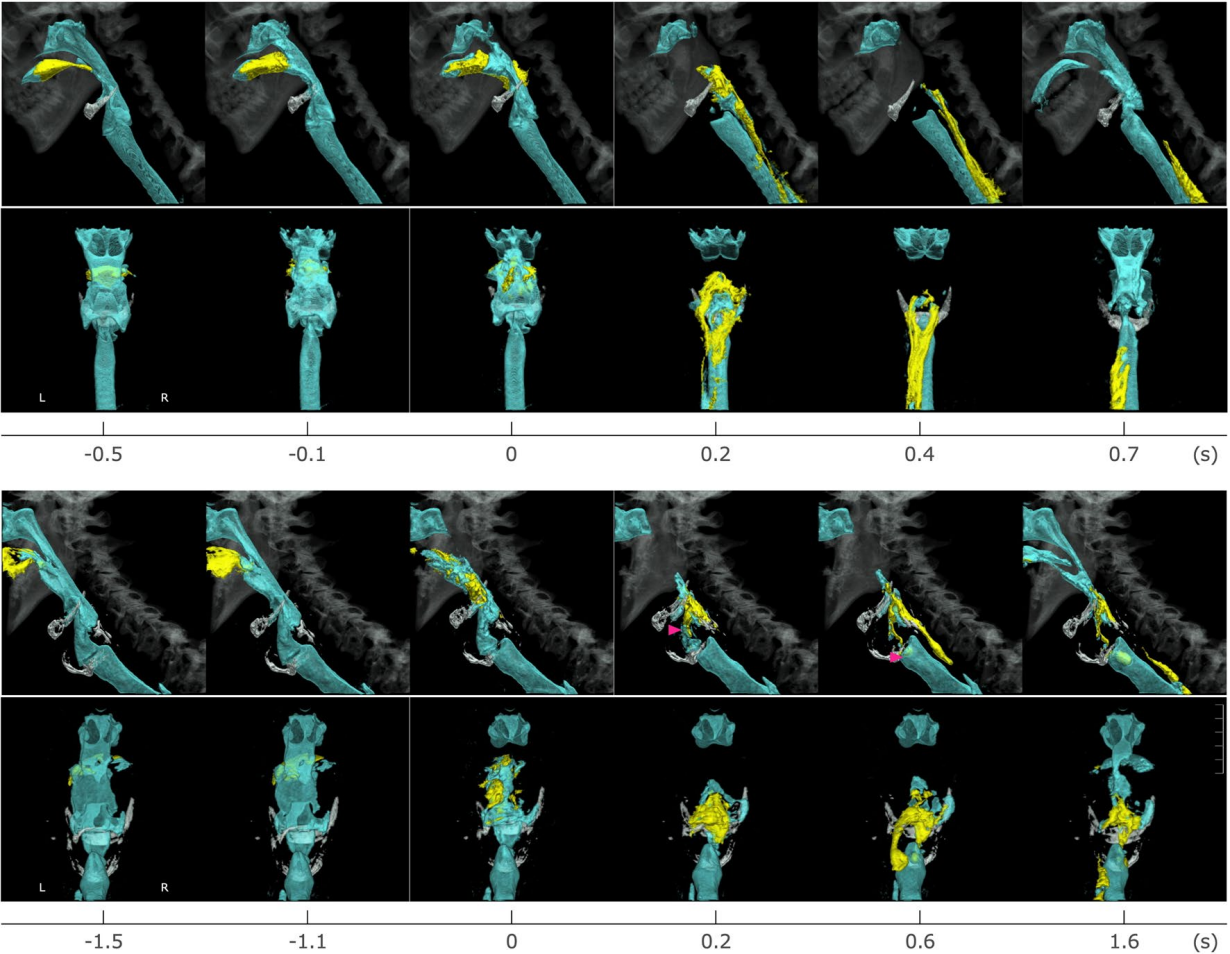
3D-CT images of swallowing in healthy volunteer (upper two rows) and in dysphagia patient (lower two rows). In both swallows, upper row is lateral view and lower row is posterior view. Yellow is bolus and blue is air column surface. Arrows show the penetration and aspiration

For image reconstruction, a half-reconstruction technique and overlapping reconstruction are used to compensate the temporal resolution. Half-reconstruction allows images to be generated from 0.1375 s of data (half the time of each tube rotation). Overlapping reconstruction allows the use of 0.1375 s data at intervals of 0.1 s by overlapping the 0.0375 s previous data. Multi-planar reconstruction images (MPR) and 3D-CT images are created from 0.5 mm thick axial images using the scanner’s software. MPR images are generated by layering 0.5 mm thin axial slices and displaying the cross sections of the structure of interest. The excellent spatial resolution and the ability to zoom and rotate MPR images are important for the study of swallowing. 3D-CT images are created using a volume rendering technique where bones, air, and contrast medium can be accurately visualized. Continuous replay of 3D-CT images produces swallowing movies (Fig. 3).

#### Position

**Fig. 4:**
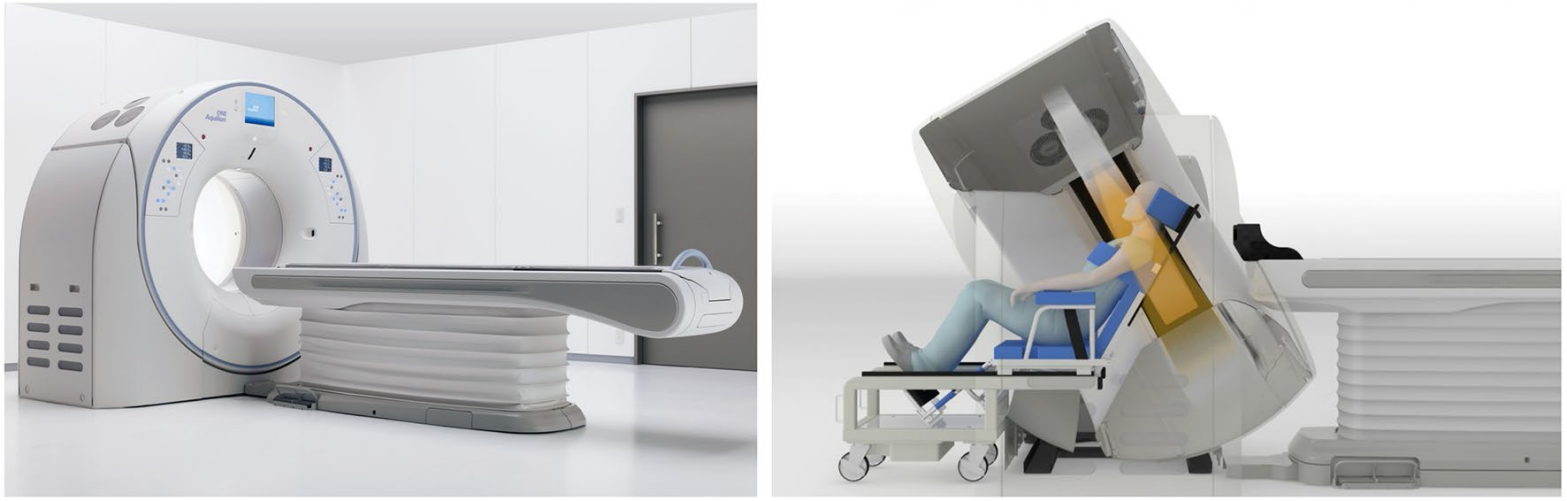
Aquilion ONE (Canon Medical) and reclining position in 320-row area detector computed tomography (320-ADCT) using offset-Sliding CT Chair. Left: Aquilion ONE Right: reclining position—30° from the upright

The typical supine position used for CT scanning was an important challenge to overcome to use this technology to study swallowing. The study of swallowing is optimally conducted upright (standing or seated at 90 degrees). To overcome this limitation, the offset sliding CT chair (eMedical, Tokyo, Japan) was designed to allow for a reclining position. This reclining chair uses a weighted-base cantilever design to allow for the seat to slide backwards and into the scanner. The chair is set on the opposite side of the CT table (back of the scanner) with the CT gantry tilted at the maximum allowed angle (22°–30° depending on the scanner) toward the chair and the chair reclined (30°–45° from the upright position) (Fig. 4).

#### Scanner Parameters

The field of view is set to 240 mm with a tube voltage/current 120 kV/40 mA. Third generation scanners parameters differ and include: 120Kv, 30 mA, 0.275 s/rotation, and 30° tilt. Oral contrast is needed for the bolus to be successfully identified in dynamic 3D-CT images. Similar to VF, barium or iodine contrast is used. The target concentration of contrast is 5–7% weight/volume depending on bolus consistency. This concentration is less than commercially available barium product in the US (i.e. Varibar^®^). At this concentration the ability to visually distinguish the bolus, air, bone, and soft tissues is optimal.

#### Radiation Exposure

The problems associated with increased radiation exposure due to diagnostic CT have been previously described [27]. Generally speaking, better quality images require more radiation exposure. Therefore, optimization of scanning parameters is extremely important to decrease radiation dose while maintaining image quality. The first radiation estimates during 320-ADCT for swallowing were reported by Kanamori and colleagues [28]. The effective dose was 3.9 mSV for the patient and 0.002 mSV for the operator. In comparison, this dose is 4 times the exposure received during VF (~ 1.0 mSV), comparable to a typical neck CT (2–3 mSV), and about half of a chest or abdominal CT (5–7 mSV). Radiation dose has been remarkably reduced with the technological developments of the second generation and third generation scanners. The second generation 320-ADCT, equipped with adaptative iterative dose reduction 3D (AIDR 3D), allows a 25% decrease in radiation dose [29]. The third generation 320-ADCT scanner (2016), equipped with a forward-projected model-based iterative reconstruction solution (FIRST), allows a 34% reduction compared to the second generation and a 51% reduction compared to first generation scanners [30]. The effective dose for patients is estimated at 1.9 mSv [30].

In summary, Swallowing CT is possible with 320-ADCT in a reclining position, 30°–45° from upright. Sequential volume data are obtained at a rate of 10 frames per second with a slice thickness of 0.5 mm at any arbitrary section. Radiation dose for one swallow is around 1.8 times more than that of 5 min-VF.

### Methodology of Swallowing CT

#### Advantages of Visualization

The first study documenting the feasibility of using 320-ADCT to obtain dynamic 3D-CT images of swallowing was published in 2011 [3]. The technique allowed 3D visualization and quantitative evaluation of swallowing. The images represent isotropic volume data with almost equal coronal, sagittal, and axial resolution (0.47 mm × 0.47 mm × 0.59 mm) thus, structures can be accurately evaluated at any arbitrary cross section. A study comparing the relative error in distance measurement between two points in adult skull specimens measured by VF and 320-ADCT demonstrated that the error of 320-ADCT was at most 0.34%, much smaller than VF [28].

**Fig. 5:**
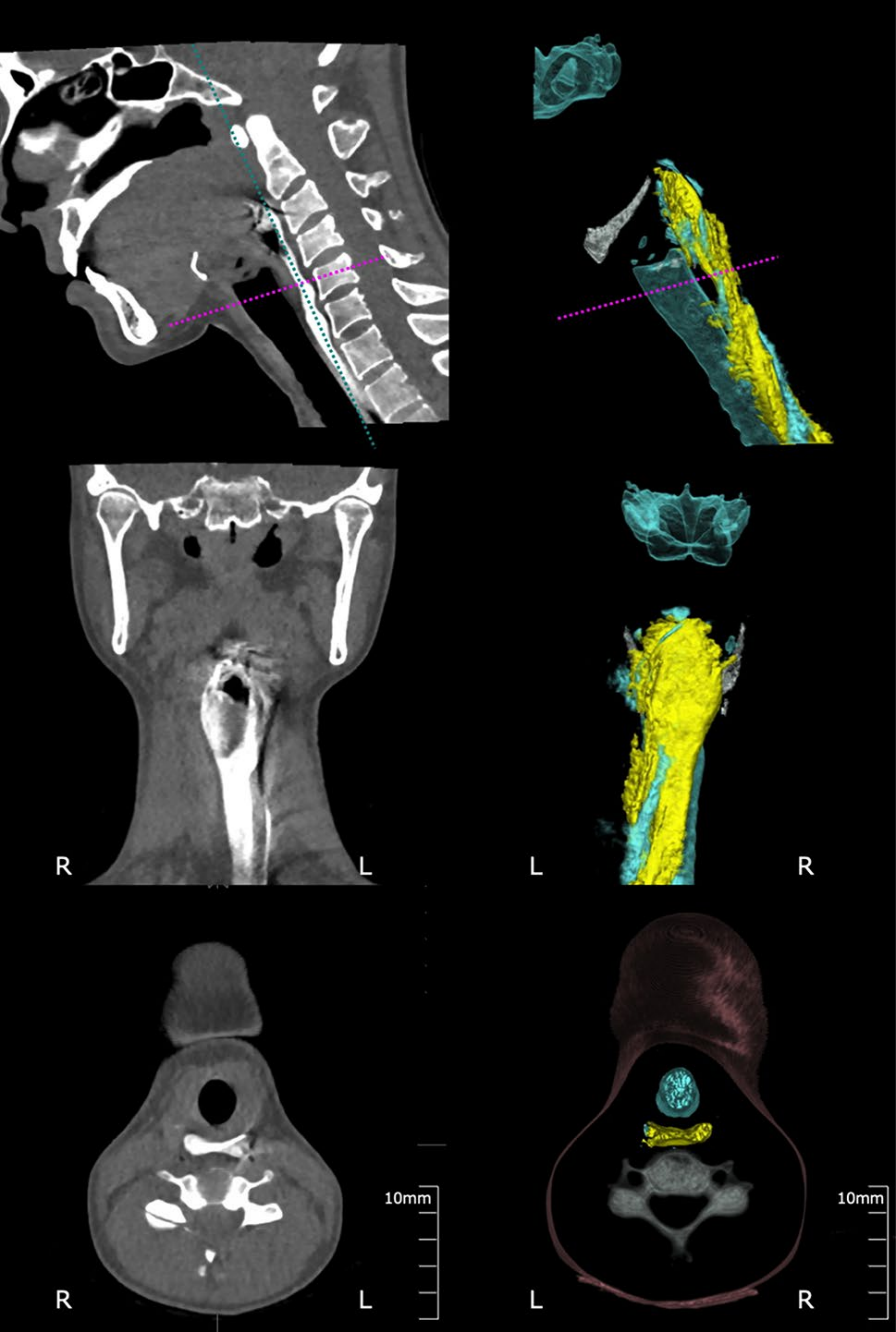
MPR images and 3D-CT images of Upper esophageal sphincter from various perspectives. Left: MPR images (upper: mid-Sagittal section, middle: coronal section, lower: axial section). Right: 3D-CT images (upper: lateral view, middle: posterior view, lower, superior view)

Simultaneous visualization of all swallowing-related structures without restriction and in motion is possible. This view of structures and motion is unique to Swallowing CT and is not otherwise available. For example, true vocal cords closure, the last line of airway defense during swallowing, can be viewed on a plane independent from other simultaneous events such as laryngeal vestibule closure. Upper esophageal sphincter (UES) motion can be viewed and evaluated from various perspectives (e.g. AP, lateral, and medial) (Fig. 5).

#### Temporal Measurements

The follow-up study reported the methodology to obtain temporal measurements of swallow related structures including hyolaryngeal movement, velopharyngeal closure, laryngeal closure, upper esophageal sphincter opening, and bolus movement. The 320-ADCT images allowed for the evaluation of the events associated with laryngeal closure (i.e., true vocal cords closure, laryngeal vestibule closure, and epiglottis inversion) separately. UES opening can be also measured accurately on axial images. This paper documented the temporal relationship between the three components of laryngeal closure and other swallowing events when swallowing 10 ml of honey-thick liquid [31]. The methodology was shown to be reliable between raters. The onset and termination of all swallowing events had high concordance as evidenced by a high interclass correlation, average of 0.98 [32].

#### Kinematic Measurements (Fig. 6)

**Fig. 6:**
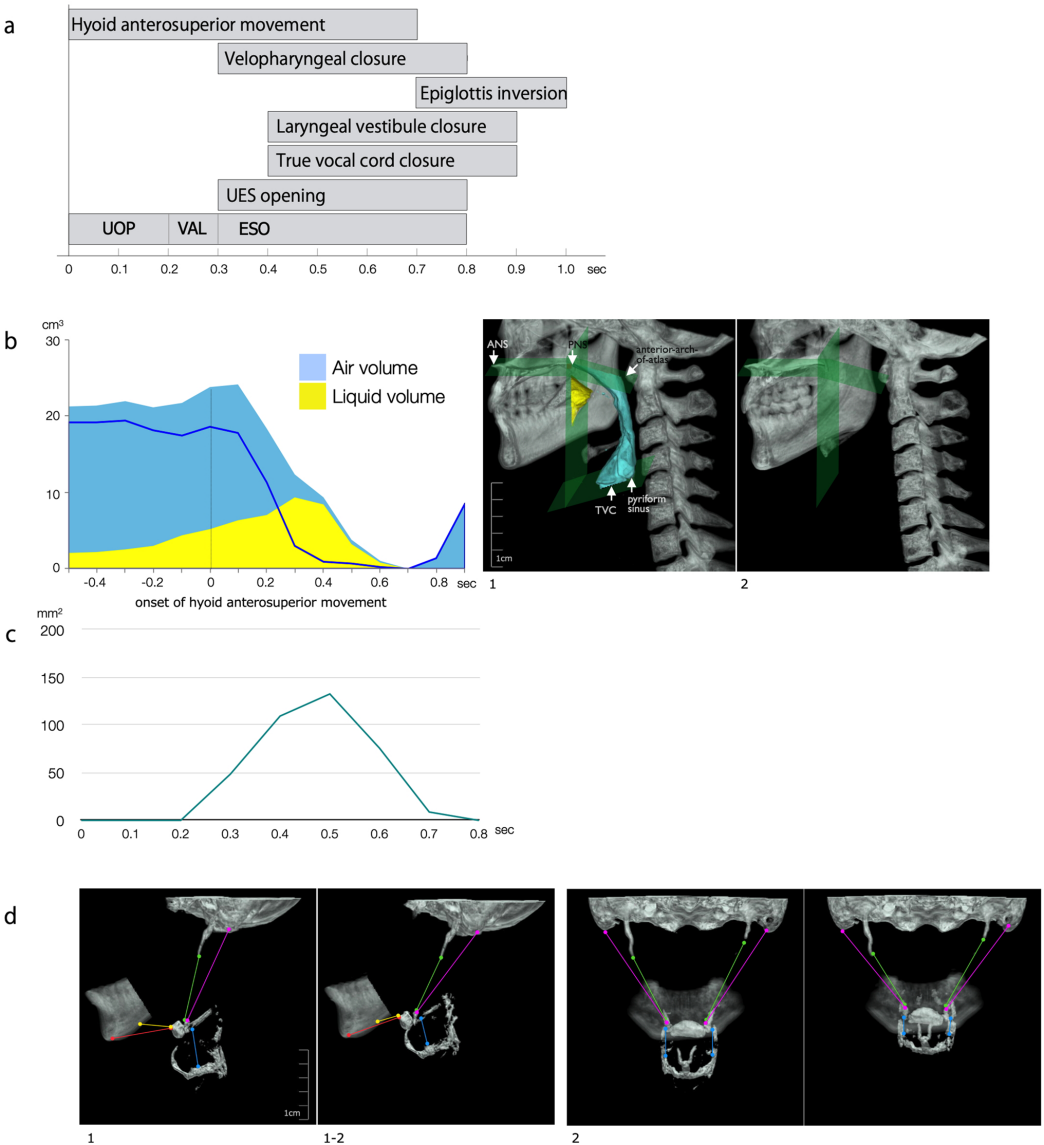
Temporal measurement and Kinematic measurements. a Temporal measurement: timing of events correlated with onset of rapid hyoid antero-superior movement as time zero. *UOP* upper orpharyngeal, *VAL* Valleculae, *ESO* esophagus. b Measurement of Pharyngo-laryngeal volume and bolus volume in pharyngeal cavity. Left: volume of air (blue line) and liquid barium (yellow) in the pharynx over time. The onset of rapid hyoid antero-superior movement is the zero reference time (vertical line). Right: 3D-images of pharyngeal cavity at the beginning of the swallow (1) and at the maximum pharyngeal contraction (2) according to the definition of pharyngeal cavity. c Measurement of the UES cross-sectional area. Cross-sectional area of UES while UES is opening. The onset of rapid hyoid antero-superior movement is the zero reference time. UES started to open 0.3 s after the hyoid antero-superior movement and was opened for 0.5 s. d Measurement of the distance between origin and insertion of the suprahyoid muscles and thyrohyoid muscles. Lateral (1) and anterior (2) views of mandibular bone, hyoid bone, thyroid cartilage, and cranial bone are illustrated. For both 1 and 2 left images are at the beginning of swallow and right images are at maximum hyoid displacement

Kinematic parameters were described later. Iida and colleagues introduced the pharyngo-laryngeal volume measurement [33]. The study described pharyngo-laryngeal volumes of approximately 20–25 ml, becoming nearly zero during pharyngeal contraction, and returning to the original volume after airway opening. Measurement of the UES cross-sectional area including antero-posterior and lateral diameter and areas, were reported later [34]. The UES was evaluated on axial images, clearly allowing for the description of its oblong shape. CT images also allowed the measurement of hyolaryngeal displacement and indirect evaluation of muscle length between origin and insertion at the hyoid [35]. The first descriptions highlighted the geniohyoid as a prominent contributor to hyoid bone anterior movement during swallowing while other muscles contributed to initial hyoid elevation.

## Morphological and Kinematical Findings

### Morphology

#### Anatomy: Pharynx and Larynx

Anatomy of the oropharynx and larynx was previously described using cadaveric, stereoendoscopic, and radiographic studies. However, due to the limitations of these methods, reported values showed large differences and the sources of the variability remained unclear. Using 320-ADCT single-volume scans, three-dimensional images were used to obtain reference values of the pharynx and larynx from 54 healthy subjects (30 male, 24 female), ages 41 ± 15 years old, and whose height was 170 ± 5 cm in males and 156 ± 5 cm in females [36] (Table 1). Bivariate analysis showed that all the length and volume measurements of the pharynx and larynx were significantly larger for men compared to women and increased with height (*p* < 0.05) [36]. Multiple regression analyses revealed that the interaction of sex, height, and age provided a more clear understanding of the configuration of larynx and pharynx [36]. Length measurements of the larynx (vertical length of larynx and the length of vocal cords) were significantly larger in men after adjusting for height. As for the effect of age, the volume of the laryngeal and hypopharyngeal cavities decreased with age in females [36]. These suggest that sex, height, and age have independent and interacting effects on the morphology of the pharynx and the larynx.

**Table 1:**
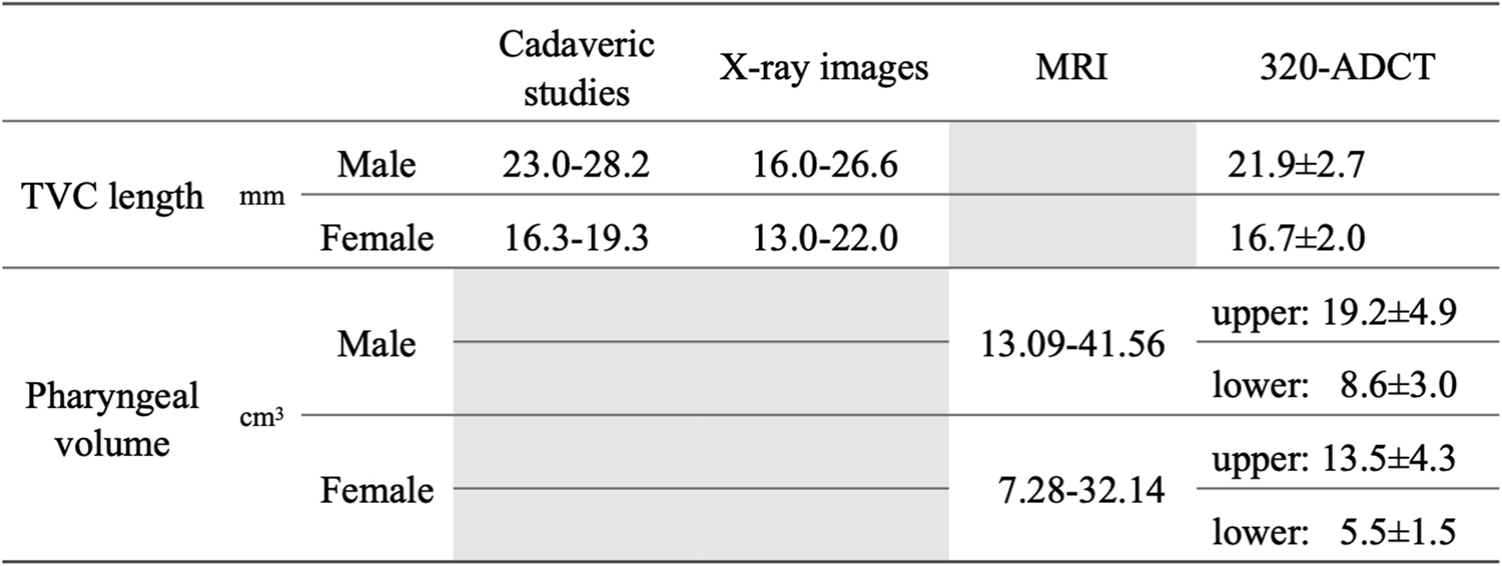
Values of TVC length and pharyngeal volme from the studies of Cadaveric, X-ray, MRI, and 320-ADCT

### Kinematics

#### Timing of Swallowing Events with the Effect of Bolus Consistency, Size, Age, and Posture

Several studies using 320-ADCT for the temporal measurement of swallowing events have been performed [37–40]. They seek to understand the motor control system, particularly airway protection, and to identify adaptable structures in different conditions, including bolus consistency, bolus size, age, and posture.

**Fig. 7:**
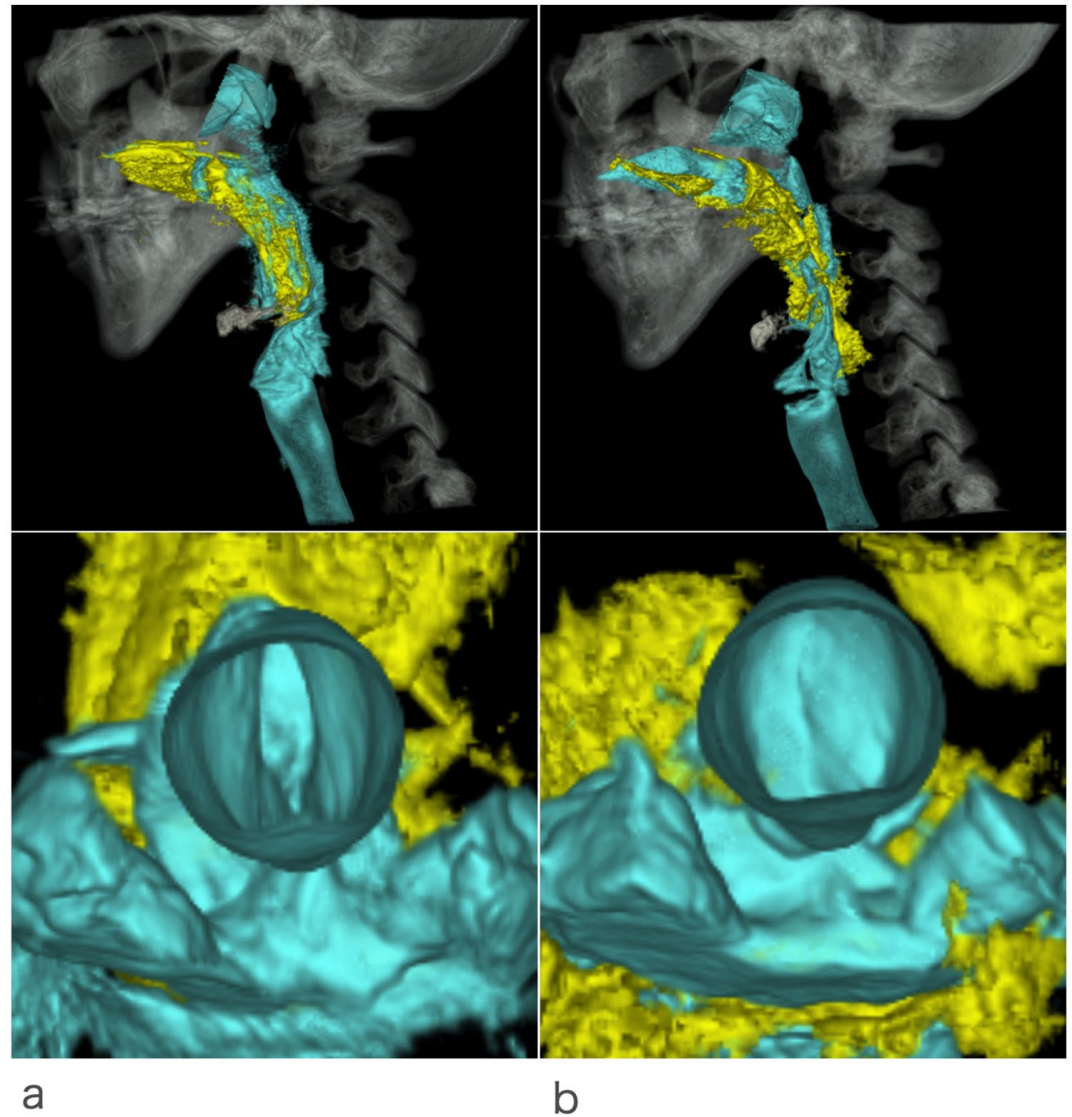
3D-CT images of one healthy volunteer’s swallow of honey thick liquid and thin liquid. Lateral image (upper) and transverse image (view of true vocal cord from below) (lower) at the onset of hyoid anterosuperior movement during honey thick liquid (**a**) and thin liquid (**b**). At the onset of hyoid anterosuperior movement, honey thick bolus was transported to valleculaetrue and vocal cord (TVC) was still open. Meanwhile, thin liquid bolus was transported to hypopharynx, lower than that of honey thick, and TVC was closed already

A study comparing swallowing of two different liquid consistencies (10 ml honey-thick liquid and thin liquid) identified that, when swallowing thin liquids, the true vocal cords closed early and remained closed longer [37] (Fig. 7). Conversely, laryngeal vestibule closure and epiglottis inversion did not vary between consistencies suggesting that true vocal cords closure occurs independent from the other two events. True vocal cords modulation was further analyzed in a study of posture and consistency [38]. The study compared the timing of events when swallowing under three different conditions: thin liquid reclining 45°, thin liquid reclining 60°, and honey-thick liquid reclining 60°. In this study it was described that at 60°, true vocal cords closure occurred earlier when swallowing thin liquids compared to thick liquids. Additionally, greater variability was noted for onset of true vocal cords closure when swallowing thin liquid reclining 45°, as 30% of subjects closed the true vocal cords before onset of swallowing. This pre-swallow true vocal cords closure suggested that the effect of gravity on the oral cavity was greater when swallowing thin liquid reclining 45°, which increased the perceived risk of airway invasion before swallowing and elicited anticipatory adjustment in true vocal cords closure.

Interestingly, true vocal cords closure changes were not identified when comparing three different honey-thick liquid volumes (3 ml, 10 ml, 20 ml) [39] nor in a study evaluating swallowing of 10 ml honey-thick liquid between three age groups [40]. A thick bolus is more cohesive and is likely perceived as less likely to cause aspiration thus true vocal cord closure is not markedly changed.

Those studies suggest that true vocal cords closure is responsive to oral sensory input based on bolus consistency. This has important implications in swallowing rehabilitation as true vocal cords closure can be targeted independently.

#### Kinematic of Swallowing

Studies using 320-ADCT have clarified the physiologic changes occurring in the pharyngo-laryngeal cavity. During usual swallowing, the pharyngo-laryngeal cavity is at its maximum volume around swallow onset, it is completely obliterated during the swallow, and then returns to rest regardless of bolus volume or consistency [33, 41]. The maximum pharyngeal volume (air volume and bolus volume) was significantly larger as the bolus volume increased (3 ml: 22.0 ± 5.7 ml, 10 ml: 23.5 ± 5.7 ml, 20 ml: 26.0 ± 4.5 ml; *p* = 0.003) [33]. The pharyngo-laryngeal volume becomes basically zero regardless of bolus size, when pharyngeal constriction against the tongue base occurs to transport the bolus into esophagus.

Quantitative analysis of pharyngeal volume has also allowed the description of bolus propulsion through the oral cavity to the UES during swallowing. Typically, the bolus and air are swallowed simultaneously; this can be seen during UES evaluation with 320-ADCT. The amount of air crossing the UES increases as the bolus volume increases [33]. The air volume was larger when swallowing thin liquids compared to thick liquids [41]. Differences in tongue and pharyngeal motion as a result of different consistencies or volumes are likely affecting the subsequent pharyngeal stripping action necessary to propel the bolus allowing for changes in the volume of air swallowed [41]. Different magnitudes of viscous force between thin liquid and thick liquid were suggested to influence the bolus flow patterns through UES. A recent study reported a novel observation, annular two-phase flow (a ring of liquid around a core of air) as thin liquids passed through the UES, which is rarely seen when swallowing thick liquids [42].

The UES cross-sectional area was first described directly using traditional CT. The duration of UES opening obtained from VF studies, which increased as the bolus volume increased, was confirmed with Swallowing CT [34]. A novel finding from Swallowing CT was the shape of UES. It was oval/elliptical with medial anteroposterior narrowing, not round [33]. Maximum cross-sectional area of the UES during swallowing of honey-thick liquid was 1.41 ± 0.45mm^2^, 1.94 ± 0.57mm^2^, 2.52 ± 0.60mm^2^ for 3 ml, 10 ml, and 20 ml respectively. Increasing bolus volume resulted in significant increases in UES maximum cross-sectional area (3–10 ml, *p* < 0.01, 10–20 ml, *p* < 0.01) [34]. These studies suggest that the UES responds to larger bolus volume by increasing opening size and duration [34].

These studies of pharygo-laryngeal volume and UES area provide normative data that will be useful for understanding pathophysiology and assessing improvement in clinical settings.

## Clinical Applications and Other Uses of Swallowing CT

### Clinical Application

Normative data acquired from the aforementioned studies are refining our knowledge of swallowing physiology and clarifying swallowing pathophysiology when pharyngeal residue and aspiration occur. Normative anatomical and kinematic data also allows to analyze the effect of swallowing strategies, maneuvers, and exercises.

#### Clarification of Swallowing Pathophysiology

In clinical settings, it is difficult to determine whether pharyngeal residue is caused by decreased pharyngeal contraction and/or UES opening. Swallowing CT allows researchers or clinicians to unveil the mechanism of residue using 3D-CT images and quantitative analysis of pharyngeal contraction and UES opening. The swallowing CT treatment-oriented evaluation allows for a more precise and quantitative analysis compared to VF or VE. This contributes to identification of optimal treatment and allows us to clearly elucidate functional changes occurring after exercise as explored in one case study [26].

#### Swallowing Strategies: Posture Techniques

Single-volume analysis was used to study morphological changes in the pyriform sinus as a result of head rotation at 30°, 45°, and 60° [43]. The volume of the pyriform sinus on the rotated side decreased by 72.2 ± 23.4% (*p* < 0.01) with 45°and by 69.4 ± 22.3% (*p* < 0.01) with 60°, compared to head neutral. Meanwhile, the volume of pyriform sinus on the opposite side increased by 172.5 ± 32.0% (*p* < 0.001) with 30°, by 172.5 ± 36.2% (*p* < 0.001) with 45°, and by 187.4 ± 31.3% (*p* < 0.001) with 60°, compared to head neutral. Similarly, upper cross-sectional area of pyriform sinus on the rotated side significantly increased and upper cross-sectional area of pyriform sinus on the opposite side significantly decreased as the angle of head rotation increased (*p* < 0.001). Depth of the pyriform sinus was not affected by head rotation angle and only the opposite side showed the significant increase with the head rotation. These changes were less likely to be affected by the angle of lateral bending and flexion/extension of the head/neck accompanied by head rotation [43].

#### Swallowing Maneuvers and Exercise

Multiple dimensional quantitative analysis has started to clarify the effect of maneuvers and exercise with precision [44, 45]. The effects of the Mendelsohn maneuver were studied in healthy subjects [44]. The analysis of critical-event timing revealed that all events were significantly prolonged and termination was delayed (*p* < 0.05) except UES opening. Hyoid and laryngeal excursion analyses showed that the hyoid bone was positioned significantly higher at maximum displacement (*p* < 0.05). Duration and cross-sectional area of UES opening were not affected by the Mendelsohn maneuver. A new finding was significantly prolonged closure of pharynx. Changes in the kinematics of swallowing with the Mendelsohn maneuver including timing and magnitude of hyoid displacement, prolonged closure of the pharynx, and prolonged velopharyngeal and laryngeal closure suggested improvement of safety and efficiency as the main effect of Mendelsohn maneuver [44].

A study of the Tongue-hold swallow analyzed pharyngeal volume to elucidate the effect of Tongue-hold swallow on the pharyngeal cavity [45]. The study revealed variable pharyngeal volume between subjects, suggesting that subjects might not perform Tongue-hold swallow in the same manner due to non-specific instruction and unadjusted tongue protrusion length [45]. Additionally, this study demonstrated increased hyolaryngeal elevation and increased cross-sectional area of the UES when performing the Tongue-hold swallow [45].

### Confirmation of Measurements Obtained with 2D Data

Swallowing CT can also be used to elaborate and confirm findings described using 2D data. 3D volumes of pharyngeal residue were compared to 2D lateral and anteroposterior area of residue in thirteen patients with dysphagia using 320-ADCT [46]. Two 2D area measures were correlated with each other and with 3D volume [46]. Linear regression analyses allowed for volume to be described using 2D data as follows: volume = 1.24 × lateral residual area − 0.63 and volume = 0.50 × anterposterior residual area − 0.20 [46]. Volume of residue was accurately predicted by both lateral (*R*^2^ = 0.91) and anteroposterior (*R*^2^ = 0.88) residue area using bivariate regression analyses with limitations in specific cases, who had the pharyngeal residue dispersed laterally, anteroposteriorly, and superoinferiorly. The results suggested that the use of area to quantify the amount of pharyngeal residue is adequate in cases that residue is not dispersed. Swallowing CT allows the validation of the morphologic and kinematic analyses of swallowing with conventional 2D methodologies.

### Simulation

The dynamic data obtained using 320-ADCT supports the study of swallowing using computerized simulations [47–49]. Simulations calculated with 320-ADCT data have been used to study the effect saliva on liquid bolus flow [47]. The effects of cancer-related tongue volume loss on swallowing physiology are difficult to characterize, thus one study used 320-ADCT data to model the effect of volume loss on swallowing physiology [49]. The model described increased oral and pharyngeal residue associated with volume loss in the tongue base [49]. This is the first step for developing a functional simulation model that helps understand comprehensive dysphagia mechanisms and may support the prediction of outcomes after clinical treatment.

## Conclusion

Swallowing CT is a groundbreaking technology that enables visualization of swallowing three-dimensionally with excellent space resolution and with sufficient time resolution. The use of Swallowing CT has resulted in dramatic advances in the study of swallowing physiology and pathophysiology in two ways; three-dimensional visualization and quantitative analysis. Swallowing CT will serve as an important tool in both research and clinical settings to promote the understanding of complex swallowing physiology and swallowing disorders, to determine clinical management, and for follow-up after treatment. Improvements in Swallowing CT technology to reduce radiation exposure and time resolution are bringing this technique closer to the clinic. Combining high-resolution manometry data by synchronization, both kinematic and kinetic information are acquired, which will clarify swallowing physiology and pathophysiology furthermore. The opportunities afforded by the availability of dynamic 3D data continue to grow and will continue to advance dysphagia rehabilitation.
